# Treatment for sleep apnea by continuous positive airway pressure improves levels of inflammatory markers - a meta-analysis

**DOI:** 10.1186/1476-9255-10-13

**Published:** 2013-03-22

**Authors:** Aaron Baessler, Rashid Nadeem, Michael Harvey, Essam Madbouly, Amna Younus, Hassan Sajid, Jawed Naseem, Asma Asif, Hasnain Bawaadam

**Affiliations:** 1 Rosalind Franklin University of Medicine and Science, Chicago Medical School, North Chicago, IL, USA; 2 Pulmonary and Sleep Medicine, James A Lovell Federal Health Care Center, North Chicago, IL, USA; 3 Bahria University medical and dental college, Karachi, Pakistan; 4 McMaster University, Ontario, Canada; 5 University of Karachi, Karachi, Pakistan; 6 James A. Lovell Federal Health Care Centre, 3001 Green Bay Road, North Chicago, IL, 60064, USA

**Keywords:** OSA (obstructive sleep apnea), CPAP (continuous positive airway pressure), CRP (C-reactive protein), TNF-α (tumor necrosis factor-alpha), IL-6 (interleukin-6), AHI (apnea-hypopnea index), ODI (oxygen desaturation index), BMI (body mass index)

## Abstract

**Background:**

Obstructive sleep apnea (OSA) is associated with coronary artery disease (CAD). Intermittent hypoxia associated with OSA increases sympathetic activity and may cause systemic inflammation, which may contribute to CAD in patients with OSA. Treatment with continuous positive airway pressure (CPAP) has been shown to change levels of inflammatory markers. We analyzed data from published studies by a systematic meta-analysis.

**Objective:**

To asses if treatment for sleep apnea by CPAP will affect levels of inflammatory markers.

**Data resources:**

PubMed, Embase and Cochrane library.

**Methods:**

Study eligibility criteria full text English studies of adult, human subjects, addressing values of at least one of the inflammatory markers before and after CPAP treatment. We used the definition of OSA as an apnea-hypopnea index (AHI) of ≥ 5/h, reported values in mean and standard deviation or median with range.

**Participants:**

Adult, human.

**Interventions:**

CPAP treatment for OSA.

**Study appraisal and synthesis method:**

A total of 3835 studies were reviewed for inclusion, while 23 studies pooled for analysis. A total of 14 studies with 771 patients were pooled for C-reactive protein (CRP); 9 studies with 209 patients were pooled for tumor necrosis factor-alpha (TNF-α); and 8 studies with 165 patients were pooled for interleukin-6 (IL-6).

**Endpoint definitions:**

The following inflammatory markers were chosen: CRP, TNF-α, and IL-6.

**Results:**

C-reactive protein: Study level means ranged from 0.18 to 0.85 mg/dl before CPAP treatment and 0.10 to 0.72 mg/dl after CPAP treatment. Mean differences, at a study level, ranged from −0.05 to 0.50. The pooled mean difference was 0.14 [95% confidence interval 0.08 to 0.20, p < 0.00001]. There was heterogeneity in this endpoint (df = 13, p < 0.00001, I^2^ = 95%).

Tumor necrosis factor-α: Study level means ranged from 1.40 to 50.24 pg/ml before CPAP treatment and 1.80 to 28.63 pg/ml after CPAP treatment. Mean differences, at a study level, ranged from −1.23 to 21.61. The pooled mean difference was 1.14 [95% confidence interval 0.12 to 2.15, p = 0.03]. There was heterogeneity in this endpoint (df = 8, p < 0.00001, I2 = 89%).

Interleukin-6: Study level means ranged from 1.2 to 131.66 pg/ml before CPAP treatment and 0.45 to 66.04 pg/ml after CPAP treatment. Mean differences, at a study level, ranged from −0.40 to 65.62. The pooled mean difference was 1.01 [95% confidence interval −0.00 to 2.03, p = 0.05]. There was heterogeneity in this endpoint (df = 7, p < 0.00001, I^2^ = 95%).

**Limitations:**

Only published data. Studies pooled were mainly small, non-randomized trials.

**Conclusion:**

Sleep apnea treatment with CPAP improves levels of inflammatory markers.

## Introduction

Obstructive sleep apnea (OSA) is a common disorder affecting about 4% of middle-aged males and 2% of middle-aged females in the developed world [1] and is a significant source of morbidity and mortality [2]. OSA is characterized by recurrent episodes of upper airway collapses during sleep. These recurrent episodes of upper airway collapse usually are accompanied by oxyhemoglobin desaturation and terminated by brief arousals which result in marked sleep fragmentation and chronic excessive daytime sleepiness (EDS) [1,3]. As a result, there is an increased expression of systemic inflammatory markers, a sustained activation of the sympathetic nervous system [4], and derangement in endothelial function [5]. Many of these physiologic and biochemical abnormalities are implicated in the pathogenesis of cardiovascular and cerebrovascular diseases, as ongoing inflammatory responses play important roles in atherosclerosis [6,7]. OSA has been increasingly linked to cardiovascular and cerebrovascular disease [8,9] and many studies have shown that OSA is associated with increased cardiovascular and cerebrovascular morbidity [10-14].

The literature suggests that an inflammatory etiology, in addition to mechanical factors, may contribute to the pathogenesis of OSA, as surgical biopsies of the uvula in patients with OSA have demonstrated histological abnormalities, including subepithelial edema and excessive inflammatory cell infiltration [15,16]. Also, the overexpression of interleukin-8 (IL-8) in human bronchial epithelial cells in response to a vibratory stimulus generated by snoring has been implicated to the pathogenesis of OSA [17]. Many studies have reported that patients with OSA have increased levels of mediators of the systemic inflammatory response, including cell adhesion molecules (ICAM), coagulation factors (Factor VIII, Tissue factor), and C-reactive protein (CRP) [18-20]. Pro-inflammatory cytokines are also up-regulated in patients with OSA [21-23]. In particular, significant elevations in serum levels of tumor necrosis factor-α (TNF-α), interleukin-1β (IL-1β), and interleukin-6 (IL-6) have been seen in patients with OSA [18,24-29]. However, some studies did not show elevation of CRP in patients with OSA [30,31].

CRP is an important serum marker of inflammation. It is synthesized from the liver and is largely under the regulation of the pro-inflammatory cytokine IL-6 [32-34]. IL-6 is believed to represent the major regulator of the hepatic acute phase response [33,34]. Unlike cytokines, CRP levels are quite stable in the same individual across 24 hours and may reflect the level of inflammatory response [35].

CRP may play a direct role in the initiation and progression of atherosclerosis [36]. Its pro-inflammatory and pro-atherogenic properties have been found in endothelial cells [37], vascular smooth muscle cells [38], and monocyte-macrophages [39]. CRP levels are also associated with oxidative stress [40]. Epidemiological studies have shown that an elevated CRP level in the high-normal (0.2 to 1.5 mg/dl) range in apparently healthy men and women is a strong predictor of cardiovascular risk [41-43]. In patients with acute coronary artery disease, stable angina pectoris, and a history of myocardial infarction, higher levels of CRP are also associated with future cardiovascular events [44,45].

IL-6 is a circulating cytokine known to be secreted from a number of different cells, including activated macrophages and lymphocytes [46]. Inflammation is the main stimulus for IL-6 production, but other stimuli also exist, such as cigarette smoke [46] and adiposity [47].

TNF-α is a pro-inflammatory cytokine that has a significant role in host defense and also mediates the pathogenesis of a number of disease processes, including atherosclerosis, septic shock, and auto-immune disorders [48]. TNF-α has two transmembrane-bound receptors and soluble forms that are released by proteolysis of the cell-bound receptor under the control of other inflammatory cytokines (e.g., IL-6, IL-2, IFN-γ), T cell activation, and by TNF-α itself [48,49].

Hypoxemia results in increases in IL-6 and CRP in normal humans [50]. Sleep fragmentation and deprivation also induces an increase in cytokines that may underlie inflammatory responses, which lead to cardiovascular morbidity [20,30]. OSA results in repetitive and severe nocturnal hypoxemia and sleep disturbances [1,3,51].

Continuous positive airway pressure (CPAP) is the primary treatment for OSA [52], since it eliminates upper airway collapse during sleep and improves sleep fragmentation, daytime symptoms [53], and quality of life [54]. Evidence shows that CPAP therapy reduces cardiovascular morbidity and risk [11,55], There are many studies with small sample sizes and few with larger sample sizes which address the effect of CPAP therapy on cardiovascular profiles and serum inflammatory markers. Therefore, we performed a meta-analysis to study the effects of CPAP on the serum inflammatory markers CRP, IL-6, and TNF-α.

## Objectives

We aim to assess the effect of CPAP treatment on inflammatory markers in human subjects with sleep apnea by comparing levels of inflammatory markers before and after specified treatment in all available published studies.

## Methods

### Studies and endpoint definitions

PRISMA guidelines were followed to perform this meta-analysis. PICOS format was followed; P: inflammatory markers (CRP, TNF-α, and IL-6), I: CPAP treatment, C; levels of markers before and after treatment period, O: decrease in marker levels. Inflammatory markers were chosen based on a review of the literature. The following inflammatory markers were chosen: CRP, TNF-α, and IL-6. Inclusion criteria for the subsequent study selection were as follows: 1) the study must have been in English; 2) full text manuscripts had to be available; 3) the study must have reported values for at least one of the markers of interest, both before and after CPAP treatment (4 weeks to 12 months after beginning treatment); 4) OSA was strictly defined as AHI of ≥ 5/h measured by polysomnography; 5) the study must have reported values in mean and standard deviation or median with range; 6) the patient number for all groups must have been reported; 7) the study must have been performed on adult (>18 years of age) humans.

### Data source and study selection

Studies for review were found by searching the PubMed and Cochrane databases with a duration from January 01, 1960 to December 31^st^, 2011. Embase was also searched with the same criteria in order to identify additional studies. Unpublished data from scientific meetings were not searched, since most abstracts do not provide enough data needed for meta-analyses. Searches were conducted using different combinations of the following key words: sleep apnea, inflammatory markers, C-reactive protein, tumor necrosis factor-α, interleukin-6, continuous positive airway pressure, autoadjusting positive airway pressure, and pressure therapy. In order to ensure that relevant sources were not left out, each marker or therapy was searched in its abbreviated form using the same word combinations as before. Multiple authors individually searched for and scored manuscripts for inclusion. Manuscripts were scored in duplicates, and if a manuscript was scored differently by two authors, then that manuscript was reviewed by a third author to finalize inclusion. The quality of studies was ranked according to the Sackett et al’s hierarchy of evidence [56] (Table 1).

**Table 1:** Quality of evidence: number and level of evidence of peer-reviewed and published papers

**Hierarchy of evidence**		
**Level of evidence**	**Description**	**No. of studies**
**1 a**	Systemic review (with homogeneity*) of randomized, controlled clinical trials (RCTs)	0
**1 b**	Individual randomized controlled clinical trial (RCT) (with narrow Confidence Interval‡)	0
**1 c**	All or none case series	0
**2 a**	Systemic review (with homogeneity*) of cohort studies	0
**2 b**	Individual cohort study (including low quality RCT; e.g., <80% follow-up)	0
**2 c**	“Outcomes” Research; Ecological studies	0
**3 a**	Systemic review (with homogeneity*) of case–control studies	0
**3 b**	Individual Case–control Study	23
**4**	Case-series (and poor quality cohort and case–control studies)	0
**5**	Expert opinion without explicit critical appraisal, or based on physiology, bench research or “first principles”	0
**Others**	Letters to editor, Abstract	0

### Data extraction and statistical analysis

Data was extracted from each study by a single author and then reviewed by a second author to ensure that no errors were made. Serum levels of inflammatory markers before and after CPAP treatment were extracted from studies as the mean with standard deviation. For studies in which data was reported in median and interquartile range, mean and standard deviation were calculated utilizing methods described by Hozo et al. [57].

Only our target variables (inflammatory markers) were recorded since we did not plan to do subgroup analyses or meta-regression. Studies that used CPAP or APAP were included in our review. If studies included data from both CPAP and APAP treatment, the each set of data was included in the meta-analysis as a separate study. For example, Patruno et al. [58] utilized both CPAP and APAP treatments. If a study involved the removal CPAP and its effects on inflammatory markers, we excluded the study from our meta-analysis. For example, Phillips et al. [59] measured the effect of short-term withdrawal from CPAP on levels of vascular inflammatory markers. The risk for bias was assessed at a study level and an outcome level. To minimize the effect of bias by including non-compliant patients, we only included compliant patients, utilizing a compliance definition of CPAP usage ≥ 4 hours on at least 70% of nights when reported by the manuscript. We also excluded the studies if OSA was not diagnosed by measured by polysomnography. For example Kohler 2009 was excluded since OSA was defined by Oxygen Desaturation Index instead of AHI. Some studies that fulfilled our inclusion criteria had to be excluded because values of inflammatory markers were exponentially larger than the values for the same inflammatory marker in all other studies. Tamaki et al. [60], for example, measured the production of TNF-α by monocytes before and after treatment with CPAP, and the values, when converted, were 1000 times greater than the other studies measuring TNF-α levels. Intercellular adhesion molecule (ICAM) and interleukin-8 (IL-8) were not included in this meta-analysis because there were not enough studies available to performed meta- analysis. Moreover oxyhemoglobin desaturation data was not included for the same reason. For studies in which no numerical data accompanied the graphical data, the authors were contacted in order to obtain the data. Authors of one study produced two independent papers that included the same CPAP and inflammatory markers data, so we only included the data from Schiza et al. 2010 [61] and not from Mermigkis et al. 2011 [62].

Statistical analyses were done using RevMan software version 5. Pooled mean difference was calculated using a random effects model for all outcomes due to the high level of heterogeneity present. Heterogeneity was assessed by calculating the Cochrane Q statistic. I^2^ statistics were also calculated to help quantify the amount of heterogeneity. An I^2^ of the following percentages represents different levels of heterogeneity: 25-49% low, 50-74% moderate, and 75-100% high. Measurement units of inflammatory markers we used in the meta-analysis were mg/dl for CRP and pg/ml for IL-6 and TNF-α. If values of any of these markers were not reported in the same standard measurement unit we used, the values were converted to the appropriate unit. Primary principal measures were differences in means of inflammatory markers before and after CPAP treatment.

## Results

**Figure 1:**
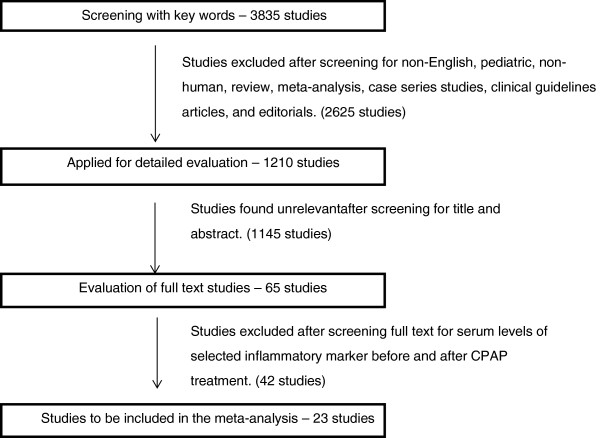
Study selection methodology.

A total of 3835 studies were reviewed for inclusion with 23 studies pooled for analysis. The quality of evidence was low (3B-individual case–control study) for all 23 studies. A total of 14 studies with 771 patients were pooled for CRP; 9 studies with 209 patients were pooled for TNF-α; and 8 studies with 165 patients were pooled for IL-6 (Figure 1). The studies measuring key serum inflammatory markers are outlined in Tables 2, 3 and 4.

**Table 2:** Selected studies measuring serum CRP before and after CPAP

**Study**	**Study design**	**Study population characteristics**	**OSA definition**	**Minimum CPAP usage**	**Outcome**
Carniero et al. 2009 [63]	Case–control	7 severely obese men	AHI > 30	≥ 5 h/day for 3 months	No significant change
Chung et al. 2011 [64]	Case–control	25 total: 22 men, 3 women	AHI ≥15	≥ 4 h/day for 3 months	No significant change
Colish et al. 2012 [65]	Case–control	47 total: 32 men, 15 women; 25 obese, 22 overweight	AHI ≥ 15	≥ 4.5 h/day for 12 months	No significant change
Dorkova et al. 2008 [66]	Case–control	16 total: 15 men, 1 woman	AHI ≥ 30	≥ 4 h/day for 8 weeks	No significant change
Harsch et al. 2004 [67]	Case–control	20 obese patients	AHI = 48.6 ± 9.1 (mean ± SD)	5.32 ± 0.67 h/day (mean ± SD) for 8 weeks	No significant change
Iesato et al. 2007 [68]	Case–control	36 total	AHI ≥ 20	≥ 4 h/day for 3 months	Significantly decreased
Ishida et al. 2009 [69]	Case–control	40 total: 32 men, 8 women	AHI > 20	> 4 h/day, 5d/week for 6 months	Significantly decreased
Patruno et al. 2007 [58] (APAP)	Case–control	15 total	AHI > 20	> 4 h/day for 3 months	Significantly decreased
Patruno et al. 2007 [58] (CPAP)	Case–control	16 total	AHI > 20	> 4 h/day for 3 months	Significantly decreased
Ryan et al. 2007 [70]	Case–control	49 total	AHI > 15	> 4 h/day for 6 weeks	No significant change
Schiza et al. 2010 [61]	Case–control	436 total: 252 men, 184 women	AHI > 15	> 4 h/day, 5d/week for 12 months	Significantly decreased
Steiropoulos et al. 2007 [71]	Case–control	20 total: 16 men, 4 women	AHI > 15	≥ 4 h/night for 6 months	Significantly decreased
Yokoe et al. 2003 [18]	Case–control	17 total	AHI ≥ 20	1 month	Significantly decreased
Zhao et al. 2011 [72]	Case–control	27 total	AHI ≥ 30	Good compliance for 3 months	Significantly decreased

**Table 3:** Selected studies measuring serum TNF-α before and after CPAP

**Study**	**Study design**	**Study population characteristics**	**OSA definition**	**Minimum CPAP usage**	**Outcome**
Arias et al. 2008 [73]	Cross-over	25 men	AHI ≥ 10	≥ 3.5 h/day for 3 months	No significant change
Carniero et al. 2009 [63]	Case–control	7 severely obese men	AHI > 30	≥ 5 h/day for 3 months	Decreased, not significant
Guasti et al. 2009 [74]	Case–control	16 total	AHI > 20	Good compliance for 12 weeks	No significant change
Minoguchi et al. 2004 [25]	Case–control	12 total	AHI ≥ 20	> 4.5 h/day for 1 month	Significantly decreased
Ryan et al. 2005 [75]	Case–control	19 total	AHI ≥ 20	4.4 h/day (mean) for 6 weeks	Significantly decreased
Ryan et al. 2006 [76]	Case–control	49 men	AHI > 5	Compliant for 6 weeks	Significantly decreased
Steiropoulos et al. 2009 [77]	Case–control	32 total	AHI > 5	≥ 4 h/day for 6 months	Significantly decreased
Tamaki et al. 2009 [60]	Case–control	33 total: 30 males, 3 females	AHI ≥ 10	3 months (no compliance data)	Significantly decreased
Vgontzas et al. 2008 [78]	Case–control	16 total	AHI > 5	≥ 4 h/day, ≥ 5d/week for 3 months	No significant change

**Table 4:** Selected studies measuring serum IL-6 before and after CPAP

**Study**	**Study design**	**Study population characteristics**	**OSA definition**	**Minimum CPAP usage**	**Outcome**
Arias et al. 2008 [73]	Cross-over	25 men	AHI ≥ 10	≥ 3.5 h/day for 3 months	No significant change
Burioka et al. 2008 [79]	Case–control	9 total	AHI > 30	5 h/day (mean) for 3 months	Significantly decreased
Carniero et al. 2009 [63]	Case–control	7 severely obese men	AHI > 30	≥ 5 h/day for 3 months	No significant change
Ryan et al. 2006 [76]	Case–control	49 men	AHI > 5	Compliant for 6 weeks	No significant change
Steiropoulos et al. 2009 [77]	Case–control	32 total	AHI > 5	≥ 4 h/day for 6 months	No significant change
Vgontzas et al. 2008 [78]	Case–control	16 total	AHI > 5	≥ 4 h/day, ≥ 5d/week for 3 months	No significant change
Ye et al. 2010 [80]	Case–control	10 total	AHI ≥ 20	> 4 h/day for 6 months	Significantly decreased
Yokoe et al. 2003 [18]	Case–control	17 total	AHI ≥ 20	1 month	Significantly decreased

### C-reactive protein

**Figure 2:**
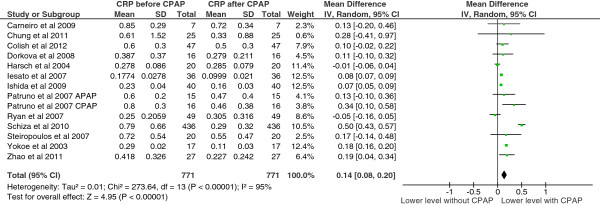
CRP levels before and after CPAP treatment.

With respect to CRP, study level means ranged from 0.18 to 0.85 mg/dl before CPAP treatment and 0.10 to 0.72 mg/dl after CPAP treatment. Mean differences, at a study level, ranged from −0.05 to 0.50. The pooled mean difference was 0.14 [95% confidence interval 0.08 to 0.20, p < 0.00001]. There was heterogeneity in this endpoint (df = 13, p < 0.00001, I^2^ = 95%) (Figure 2).

### Tumor necrosis factor-α

**Figure 3:**
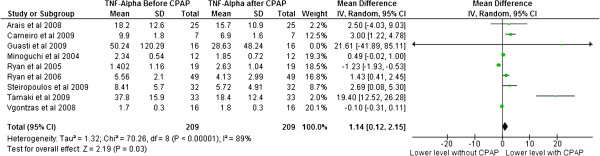
TNF-α levels before and after CPAP treatment.

With respect to TNF-α, study level means ranged from 1.40 to 50.24 pg/ml before CPAP treatment to 1.80 to 28.63 pg/ml after CPAP treatment. Mean differences, at a study level, ranged from −1.23 to 21.61. The pooled mean difference was 1.14 [95% confidence interval 0.12 to 2.15, p = 0.03]. There was heterogeneity in this endpoint (df = 8, p < 0.00001, I^2^ = 89%) (Figure 3).

### Interleukin-6

**Figure 4:**
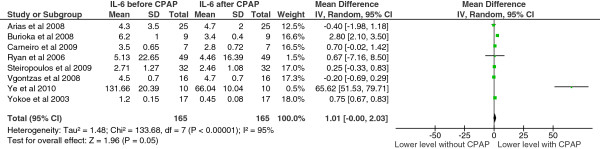
IL-6 levels before and after CPAP treatment.

Study level means ranged from 1.2 to 131.66 pg/ml before CPAP treatment and 0.45 to 66.04 pg/ml after CPAP treatment. Mean differences, at a study level, ranged from −0.40 to 65.62. The pooled mean difference was 1.01 [95% confidence interval 0.00 to 2.03, p = 0.05]. There was heterogeneity in this endpoint (df = 7, p < 0.00001, I^2^ = 95%) (Figure 4).

## Discussion

The present meta-analysis showed that CPAP therapy improves serum levels of the inflammatory markers CRP, TNF-α, and IL-6. Using a p-value of <0.05 to mark a significant change, the levels of CRP and TNF-α were significantly decreased, whereas the levels of IL-6 showed no significant change. IL-6 levels did, however, show a general trend of decreasing values with CPAP usage.

With respect to CRP, the following studies agreed that CPAP usage significantly decreases serum levels of CRP: Iesato et al. 2007 [68], Ishida et al. 2009 [69], Patruno et al. 2007 [58], Schiza et al. 2010 [61], Steiropoulos et al. 2007 [71], Yokoe et al. 2003 [18], and Zhao et al. 2011 [72]. With respect to TNF-α, the following studies agreed that CPAP usage significantly decreases serum levels of TNF-α: Minoguchi et al. 2004 [25], Ryan et al. 2005 [75], Ryan et al. 2006 [76], Steiropoulos et al. 2009 [77], and Tamaki et al. 2006 [60]. With respect to IL-6, the following studies agreed that CPAP usage significantly decreases serum levels of IL-6: Burioka et al. 2008 [79], Ye et al. 2010 [80], and Yokoe et al. 2003 [18].

We also examined why some studies did not agree with our overall finding that CPAP significantly improves levels of inflammatory markers. With regards to CRP, Carniero et al. 2009 [63] showed there was no significant change in the levels of CRP after CPAP, and this could possibly be due to the small sample population (7 subjects) of the study. Chung et al. 2011 [64], Dorkova et al. 2008 [66], Harsch et al. 2004 [67], and Ryan and colleagues [70] showed no significant change in serum CRP levels, possibly because of the small sample populations (25, 16, and 20, respectively) and possibly because there was no significant weight reduction among the patients in the study. There is some debate on whether CRP levels are dependent on obesity or the severity of OSA [31,81]. Kohler and colleagues [82] performed a randomized controlled trial and similarly concluded that 4 weeks of CPAP had no significant reduction in CRP levels, possibly due to the fact that many of the subjects also had a number of other comorbidities in addition to OSA.

Five of the nine studies measuring TNF-α levels showed that CPAP usage significantly decreases serum levels of TNF-α [25,60,75-77]. Studies that showed no significant change in TNF-α were examined to determine why those studies did not agree with our overall findings. Carniero et al. 2009 [63] had a very small sample population (7) and showed that CPAP usage over 3 months decreases serum TNF-α, though not significantly. Guasti et al. 2009 [74] also had a small sample population (only 16) and many of the patients had other comorbidities, such as elevated BMI. In addition to a small sample population (16) and patients having high BMIs, Vgontzas and colleagues [78] noted that there were discrepancies in the compliance of the patients using CPAP. Only 10 of the patients used CPAP for more than 4 hours per night.

With respect to IL-6, only three of nine studies showed that CPAP usage decreases serum levels of IL-6 [18,79,80]. The rest of studies examined showed no significant change in serum IL-6 after CPAP treatment. Again, these studies all had populations under 50 people, and the patients also exhibited comorbidities, like obesity. A few of these studies [18,76,82] only measured the effect of CPAP on systemic inflammation over 4–6 weeks, which is relatively short compared to many of the other studies examined.

There are a few limitations of this meta-analysis. It is very clear that the available literature is largely low-level evidence. Most of the studies included in the meta-analysis have examined the confounding factors (age, AHI, BMI), which we did not adjust, since we did not perform a meta-regression analysis. Moreover we did not perform the subgroups analysis to examine effect of severity of OSA on inflammatory markers before or after treatment. There are number of studies available in which levels of these markers were measured in patients with OSA and controls. Those studies cannot be included because of significant methodological differences (no CPAP). We performed a metaregression analysis on this larger pool of studies (submitted to JCSM being reviewed). Also, we did not account for CPAP compliance rates. If we had included the data from non-compliant CPAP groups, the serum levels of the selected inflammatory markers may have been affected. Another potential limitation is that we excluded all papers written in languages other than English, which could raise the possibility of publication bias. We have excluded some studies with exponentially high values when compared to the other studies in this meta-analysis. Including those studies could affect the net result of the meta-analysis. We were not able to retrieve the numerical data from some studies that reported data only in graphical form. That data inclusion could have affected the results of our meta-analysis. It is known that studies with positive results tend to get published while studies with negative results are less likely to be published, and we only included data from published studies in our meta-analysis. This could have led to publication bias as well.

Despite all these variations, it was reassuring that in majority of the studies (regardless of the composition of the study) those with CPAP treatment have lower levels of systemic inflammatory markers. This suggests that selection and sampling biases were unlikely to be responsible for the observed associations. The improvement in inflammatory markers suggests that OSA treatment modulates the cardiovascular risk profile through multiple mechanisms, including inflammation, which may play an important role for the development of atherosclerosis. Further studies are required to explore this dimension of the cardiovascular risk profile, such as the impact of OSA treatment on atherosclerosis and vasculopathy.

In conclusion, CPAP usage for patients with OSA significantly decreases serum inflammatory markers CRP and TNF-α. Also, CPAP usage seems to decrease serum levels of IL-6.

## Competing interests

All authors declare that they have no competing interests.

## Authors’ contributions

AY, HS, AA, HB, performed searches and scoring of manuscripts. RN, EM, MH, AB, and JN participated in conception of aim, scoring manuscripts and writing manuscript. AB, MH, RN, JN and AA and HB performed data extraction and data double check. RN, JN performed data analysis and interpretation. All authors read and approved the final manuscript.
